# Rare Case of Scapular Body Chondrosarcoma Treated With Limb-Salvage Partial Scapulectomy: A Case Report Highlighting Surgical Technique

**DOI:** 10.7759/cureus.71519

**Published:** 2024-10-15

**Authors:** Sara Alfadil, Falwa M Alarnous, Fahad alsuwayeh, Osama Alshaya, Khaled K AlAbbasi

**Affiliations:** 1 Executive Medical Administration, King Fahad Medical City, Riyadh, SAU; 2 Medicine and Surgery, Alfaisal University College of Medicine, Riyadh, SAU; 3 Orthopaedic Surgery, King Fahad Medical City, Riyadh, SAU; 4 Orthopedic Surgery, King Fahad Medical City, Riyadh, SAU; 5 Orthopedic Oncology and Arthroplasty/Orthopedic Surgery, King Fahad Medical City, Riyadh, SAU

**Keywords:** bone tumor, case report, chondrosarcoma, oncology, scapula

## Abstract

Chondrosarcomas are the second most malignant mesenchymal tumors that originate from neoplastic cells producing cartilage. It usually affects different sites in the body, such as the pelvis, humerus, and ribs, and rarely involves the scapula. The main modality of treatment of chondrosarcoma is surgery, while chemotherapy has been shown to be an ineffective treatment, and radiotherapy is only used in high-grade tumors. The present study reports a rare case of chondrosarcoma in a 34-year-old male patient who presented with a one-year history of a mass in the right scapula. The patient was managed by a multidisciplinary team, where surgery was the treatment of choice since the tumor was resistant to chemotherapy and radiotherapy. The postoperative pathological diagnosis was pleomorphic chondrosarcoma. The patient postoperatively received routine follow-up. Having chondrosarcoma in the scapula is rare since few studies are found in the literature. Many surgical approaches for chondrosarcoma were mentioned, but wide surgical excision has the best clinical outcome when compared with others.

## Introduction

Sarcomas are rare mesenchymal tumors that account for less than 1% of all cancer types [1]. These tumors originate in the cartilage, bone, and muscle tissues. They are broadly categorized into two main groups: bone sarcomas and soft tissue sarcomas. Among these, chondrosarcoma stands out as the second most prevalent malignant tumor following osteosarcoma. It is primarily composed of neoplastic hyaline cartilage produced by tumor cells [2].

Chondrosarcoma constitutes approximately 20% of all malignant bone tumors and exhibits a wide range of clinical behaviors and morphological characteristics that vary based on histological subtype. Histologically, chondrosarcomas can be classified into several categories, including conventional (the most common variant, comprising about 90% of cases), clear cell, dedifferentiated, and mesenchymal subtypes. These tumors can arise in any anatomical region where cartilage is present, including the pelvis, femur, humerus, and ribs. However, scapular chondrosarcoma is relatively uncommon, representing only 5% to 7% of all reported chondrosarcoma cases in the literature [3].

Surgery remains the primary treatment modality for chondrosarcoma, with surgical outcomes serving as key determinants of prognosis, mortality, and morbidity. Notably, chondrosarcoma is often resistant to chemotherapy and radiotherapy. While radiotherapy can be utilized as an adjuvant treatment for high-grade tumors and in cases of residual disease, it is not typically effective as a standalone therapy [1-3].

The present case report highlights a relatively rare presentation of large scapular chondrosarcoma, a significantly rare tumor, managed effectively through a limb-salvage wide surgical excision. Remarkably, the patient retained a good functional range of motion in the shoulder girdle following the complete removal of the tumor, and no postoperative complications were reported.

## Case presentation

A 34-year-old male was referred from a rural hospital to our tertiary care center with a one-year history of significant swelling in his right upper back. The tumor manifested suddenly and progressively increased in size, ultimately impairing his daily activities. The patient reported no history of trauma that could explain the sudden appearance of the mass. Despite the notable swelling, the patient did not exhibit any accompanying symptoms. He denied experiencing pain, fever, weight loss, or night sweats, which explains the reason for the delayed presentation, along with patient negligence and the wrong interpretation of the tumor by multiple rural peripheral hospitals. 

**Figure 1:**
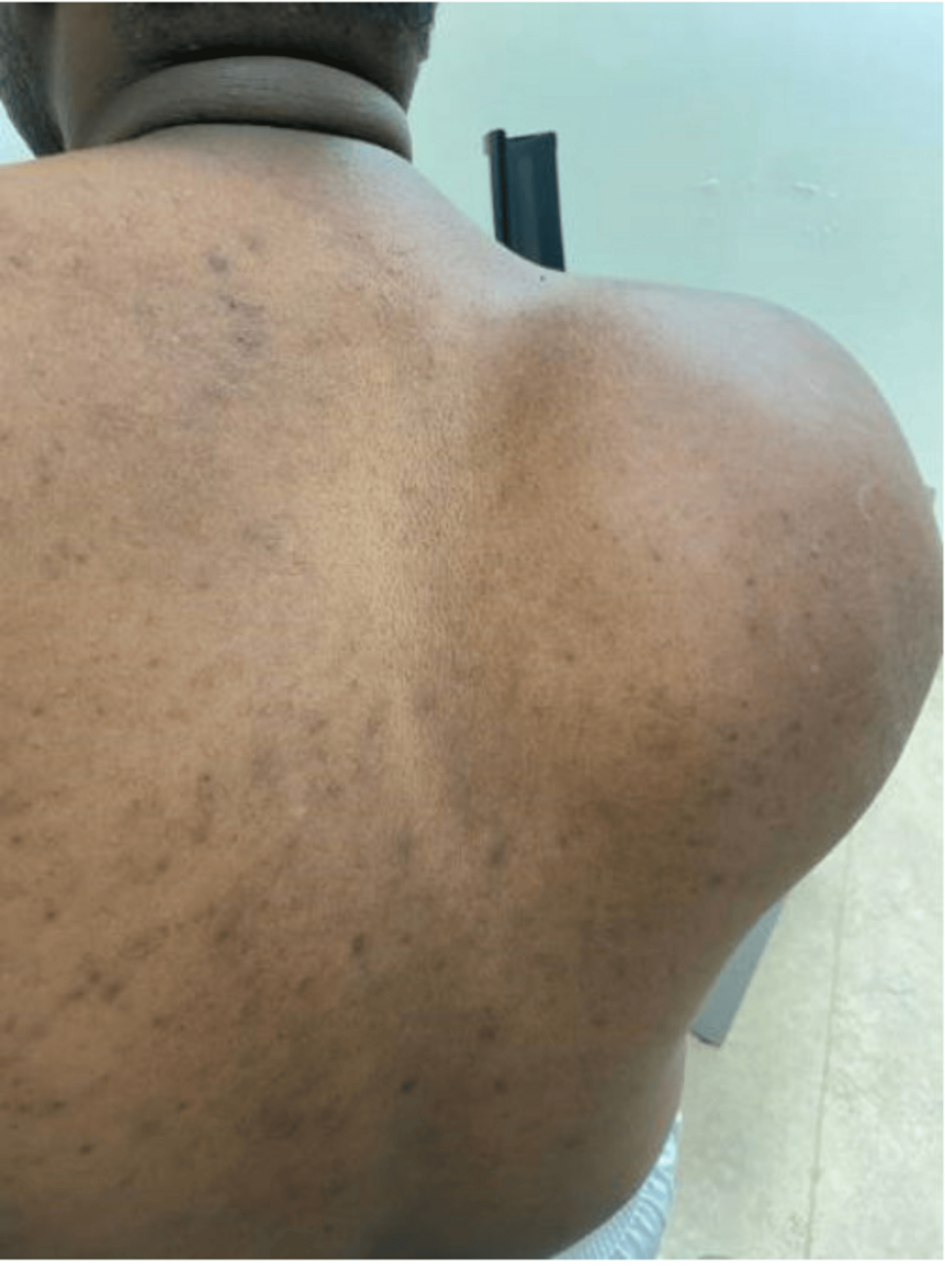
Clinical picture of the lesion involving the right posterior scapula.

Physical examination revealed a substantial soft tissue mass measuring 17.3 × 11.3 × 6.2 cm in the region of the right scapula. The mass was firm in consistency, with normal skin overlying it. Additionally, the patient demonstrated a restricted range of motion in the right shoulder; however, he retained good muscle power in that limb (Figure 1).

**Figure 2:**
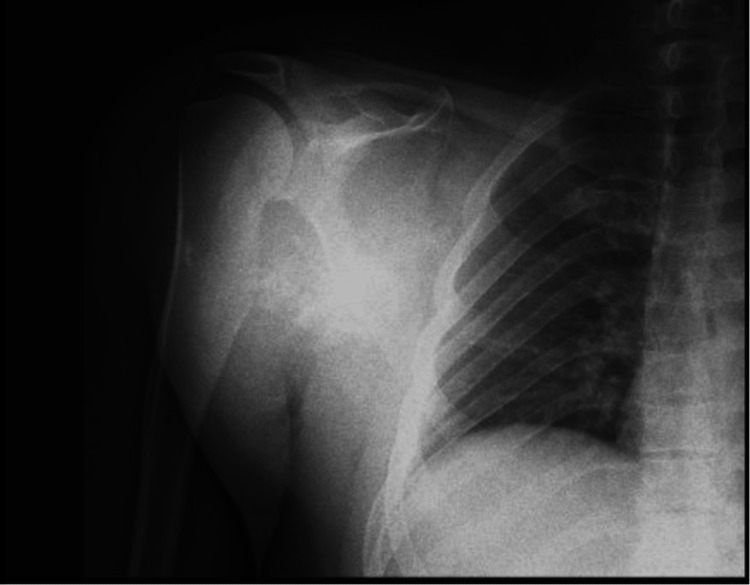
Anteroposterior preoperative X-ray view showing a large soft tissue mass along the chest wall and right axillary region.

Routine laboratory tests, including complete blood count, electrolytes, biochemical profile, and coagulation function, yielded normal results. The chest radiograph (X-ray) showed ill-defined increased haziness in the right hemothorax and right axilla due to soft tissue mass along the chest wall, extending into the right axillary region with areas of calcification (Figure 2).

**Figure 3:**
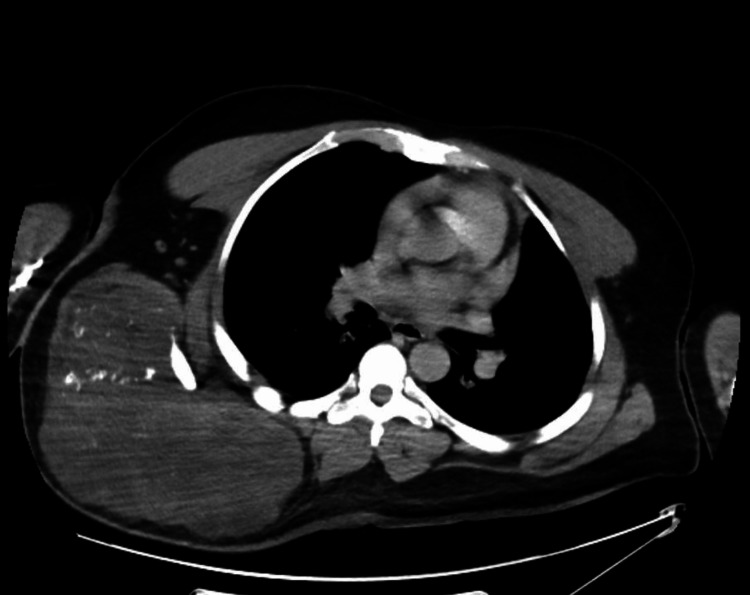
Soft tissue window axial CT view showing a large, aggressive right scapular bone lesion.

**Figure 4:**
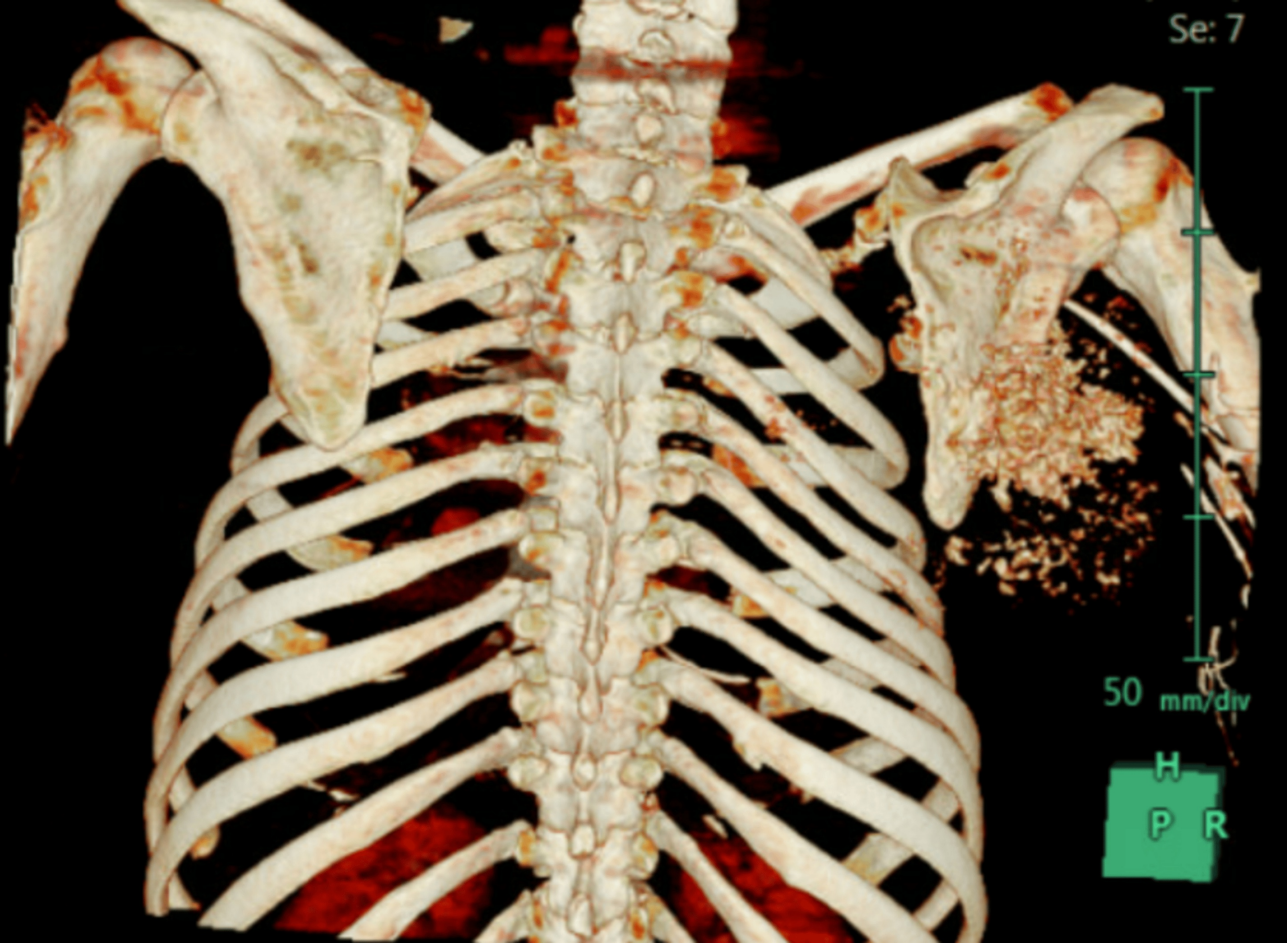
Preoperative 3D CT scan image of the scapula showing the extensive destruction of the lower two-thirds of the scapular body.

A computed tomography (CT) scan revealed a large, aggressive, and ill-defined lesion involving the right scapular body, characterized by central intense activity (Figure 3 and Figure 4).

**Figure 5:**
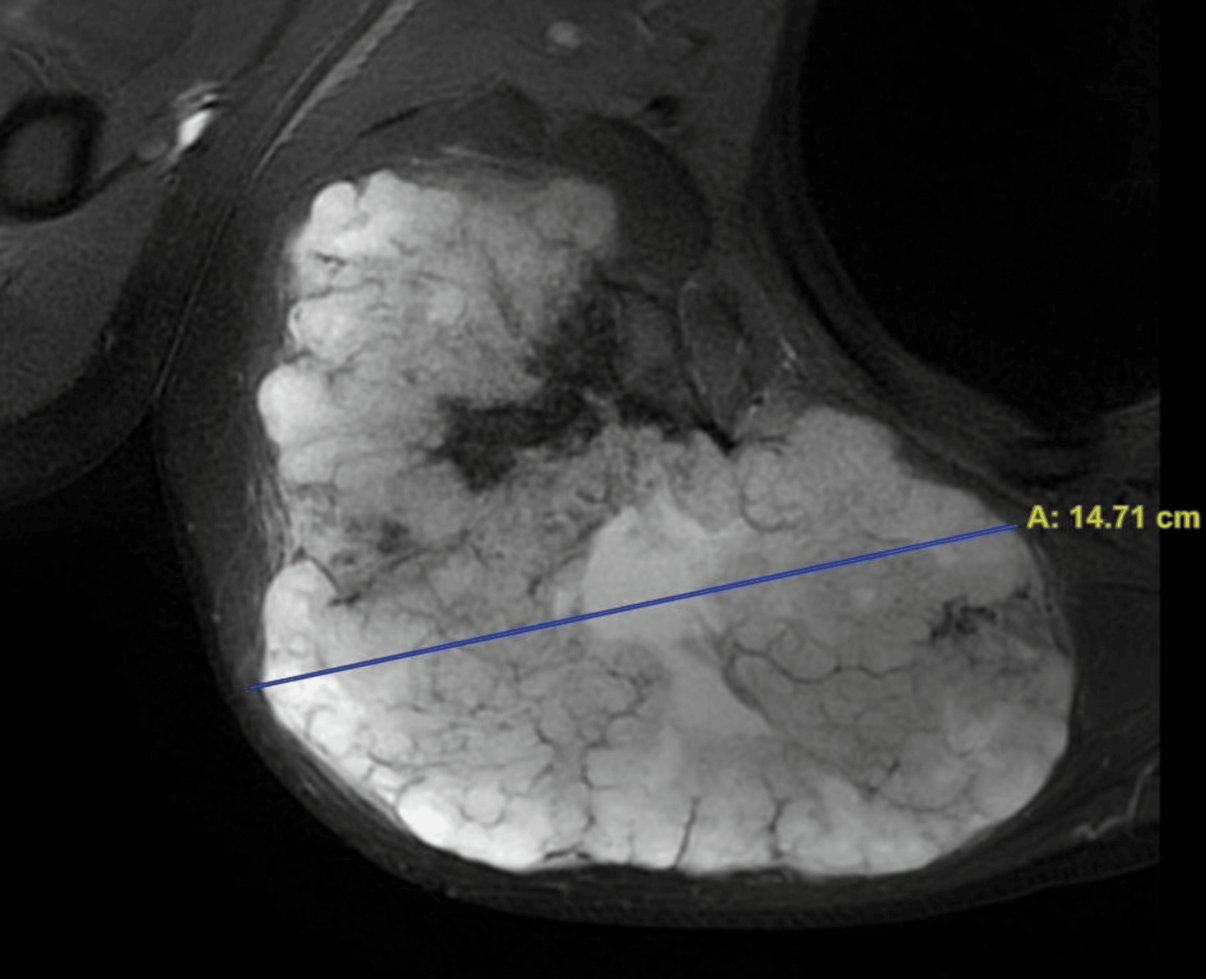
Preoperative axial MRI view. The tumor measured 14.71 cm in the maximum medial-to-lateral axis.

**Figure 6:**
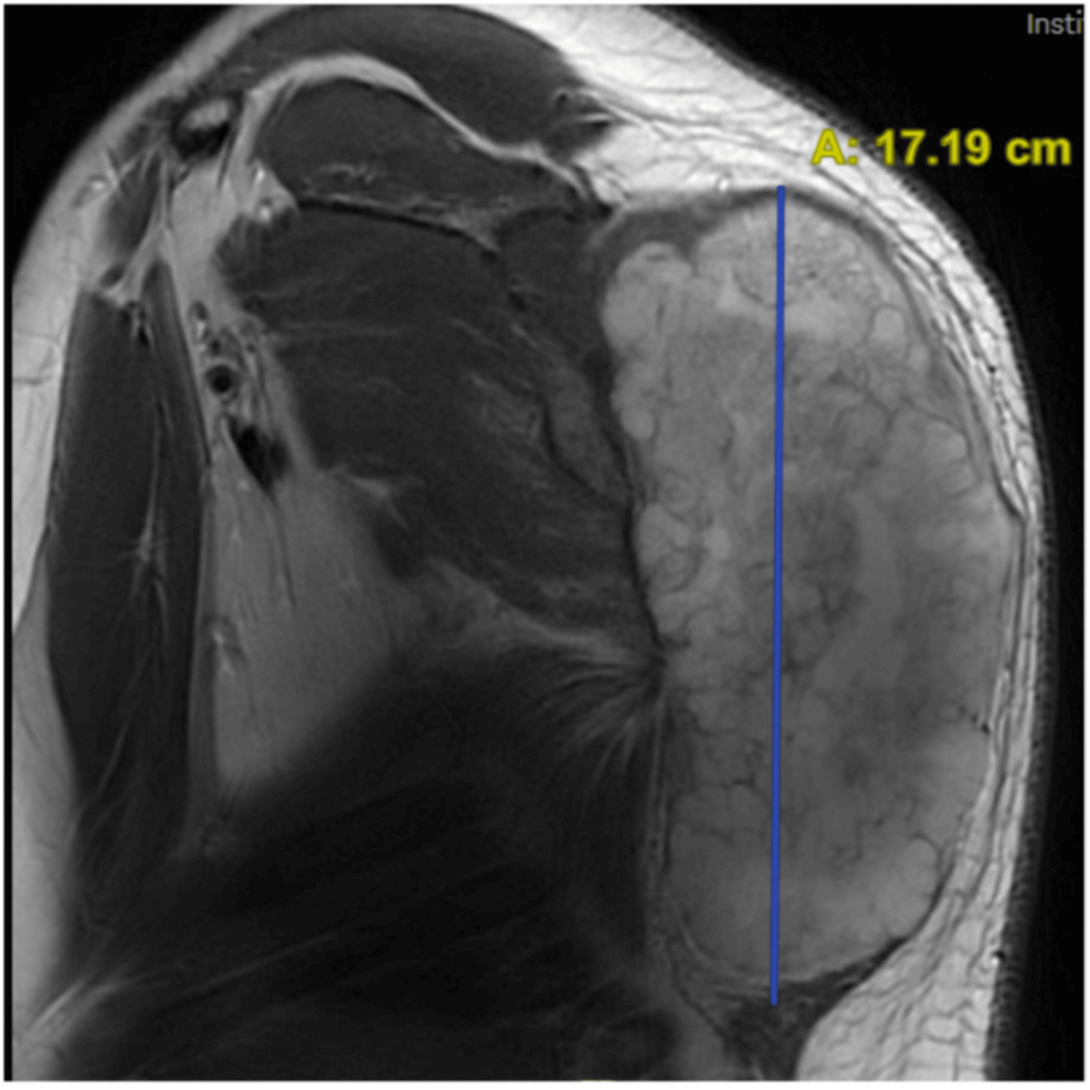
Preoperative sagittal MRI view. The tumor measured 17.91 cm in the maximum superior to the inferior axis.

A magnetic resonance imaging (MRI) study of the right scapula was done. It indicated a substantial lobulated soft tissue mass within the right upper back muscles, arising from the right scapular body, below the level of the scapular spine. This mass demonstrated high T2 signal intensity, central calcifications, and heterogeneous peripheral and septal enhancement. The lesion extended deeply in the inferior aspect, abutting the right posterolateral chest wall; however, there was no evidence of direct invasion (Figure 5 and Figure 6).

A positron emission tomography (PET) scan indicated a large malignant lesion in the scapula with a significant soft tissue component exhibiting heterogeneous mild fluorodeoxyglucose (FDG) uptake surrounding a large area of central necrosis. Prior to surgical intervention, a biopsy was performed, which revealed a hypocellular cartilaginous lesion with mild cellular atypia, nuclear binucleation, and focal myxoid changes. Importantly, there were no findings of osteoid components, necrosis, or mitotic activity.

The patient was electively scheduled for a limb-salvage wide surgical resection of the tumor. Since the large chondrosarcoma emerged from the scapular body below the level of the scapular spine, the decision was made by the two senior authors to excise the lower two-thirds of the scapular along with the overlying muscles and soft tissue. The aim was to preserve the suprascapular notch along with the supraspinatus muscle, the acromion along with the origin of the deltoid, the upper trapezius muscle inserting to the acromion and scapular spine, the glenoid, and the superior suspensory complex of the shoulder. The goal was to preserve the stability and biomechanics of the glenohumeral joint as much as possible while maintaining some functional level of the shoulder. 

Under general anesthesia, the procedure commenced with the patient positioned prone. The right upper back was prepped and draped from the medial edge of the contralateral scapula and from the base of the neck superiorly to the iliac crest inferiorly. The ipsilateral upper limb was prepped and draped in a fashion that allowed free movement of the whole limb. A posterior approach to the scapula (L-shaped incision) was employed, with the horizontal limb extending over the scapular spine and the vertical limb positioned medially to the scapula, curving distally towards the medial aspect. 

Dissection was performed in layers while maintaining meticulous hemostasis, allowing for the lateral movement of a thick flap. The lower trapezius muscle was detached from the scapular spine and flipped medially. The insertions of the levator scapulae, the rhomboid major, and the rhomboid minor muscles were dissected from the medial edge of the scapula. The origins of the long head of the triceps, the teres minor, the teres major, and the latissimus dorsi muscles were dissected from the lateral and the inferior edges of the scapula. The infraspinatus muscle was tenotomized at the musculotendinous junction, and the muscle belly was resected as an en bloc with the resected scapular body. 

**Figure 7:**
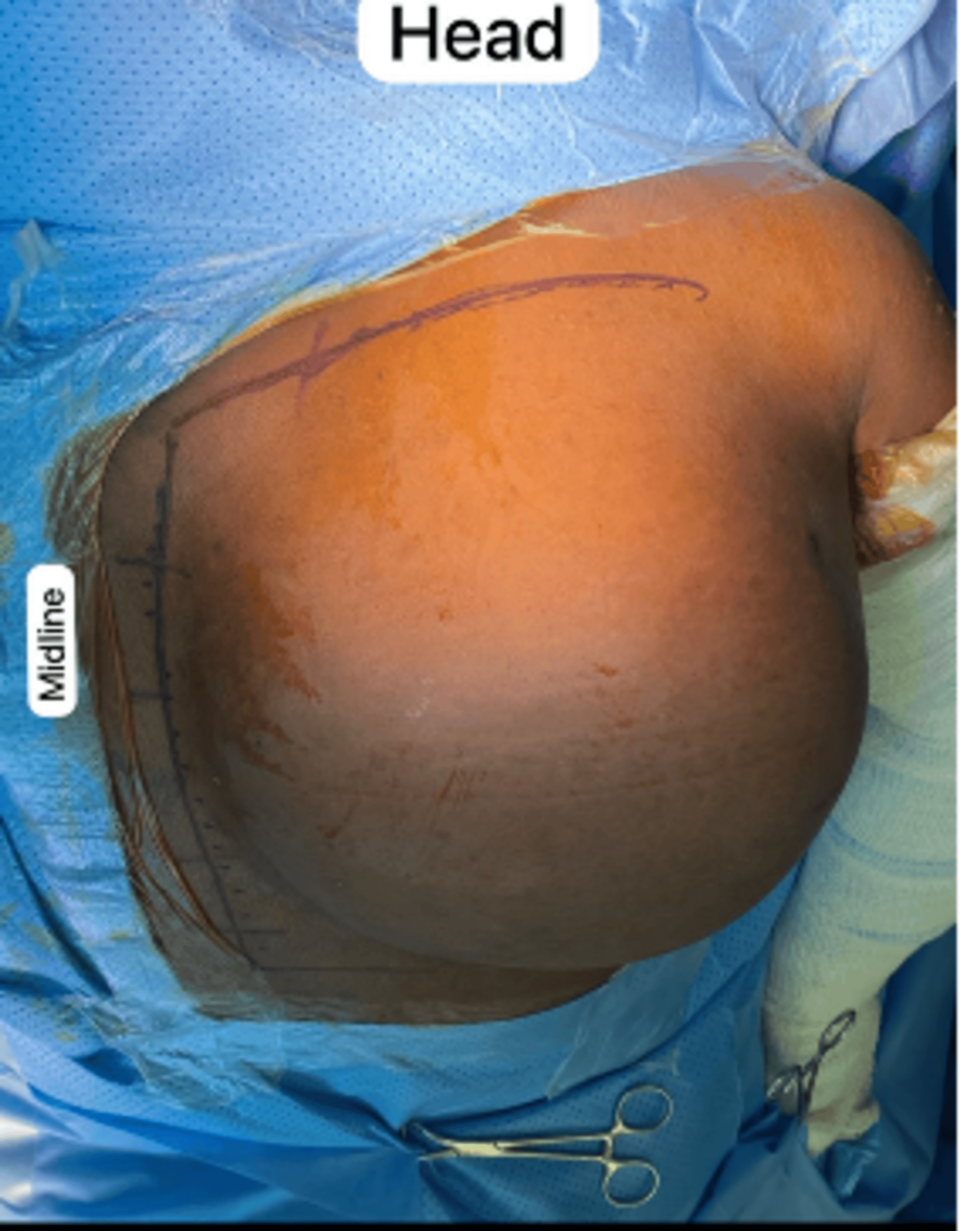
Preoperative image while the patient is prone on the surgical table. An L-shaped incision was used.

**Figure 8:**
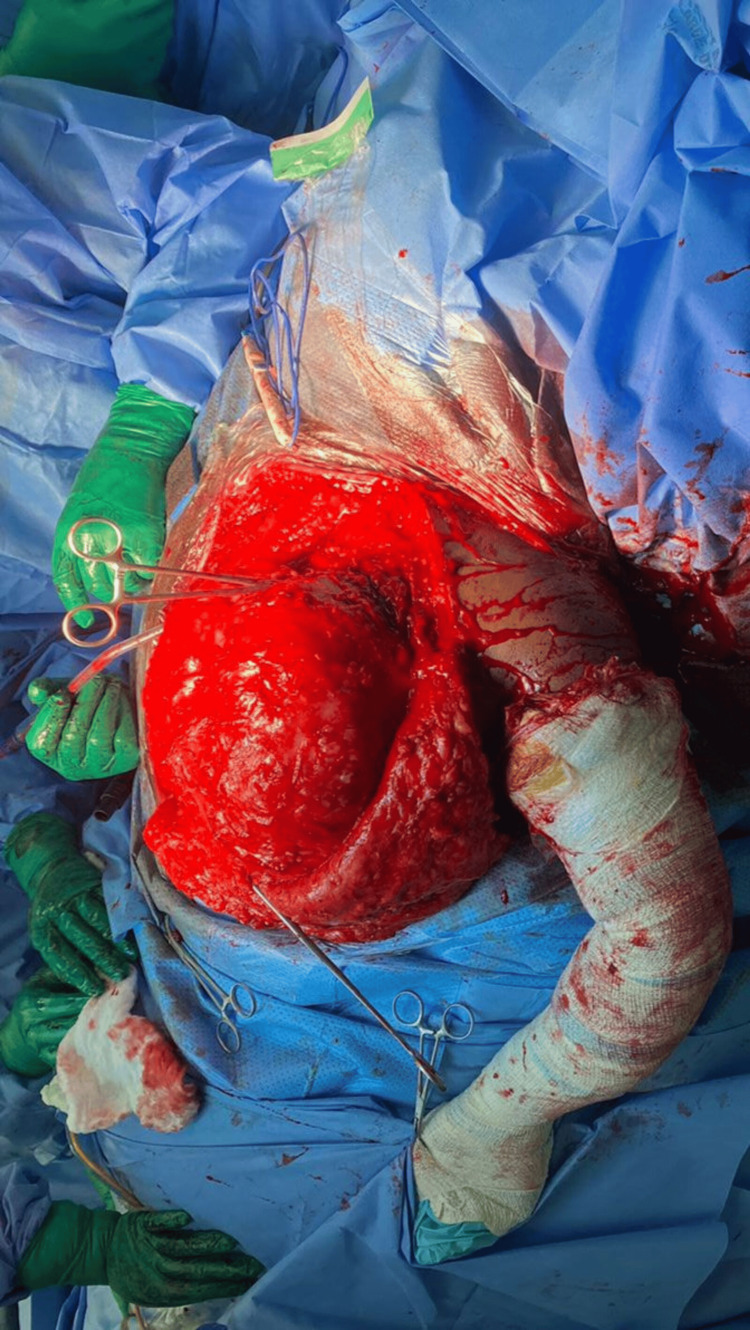
Intraoperative image showing the large chondrosarcoma tumor after raising the lateral cutaneous flap. The clamp is placed on the scapular spine.

The bone step cut was done using a small blade saw. The cut was started medially just below the level of the scapular spine and extended laterally to a point lateral to the suprascapular notch, after which the step cut was made by directing the osteotomy inferiorly for 4 cm and then laterally again to preserve the whole vault of the glenoid. The suprascapular nerve was identified and cut just after emerging from the notch. The osteotomized lower two-thirds of the scapular body was then elevated superiorly using bone hooks. The musculotendinous junction of the subscapularis muscle was then identified, and a tenotomy was done at that level (Figure 7 and Figure 8). The muscular belly of the subscapularis muscle was excised en bloc with the resected tumor. Meticulous care should be taken when elevating the scapular body and dissecting anterior to the subscapularis muscle to avoid injuring any of the nearby vitals structures within the rib cage or the vital vessels and brachial plexus found within the axilla, lateral to the scapula.

**Figure 9:**
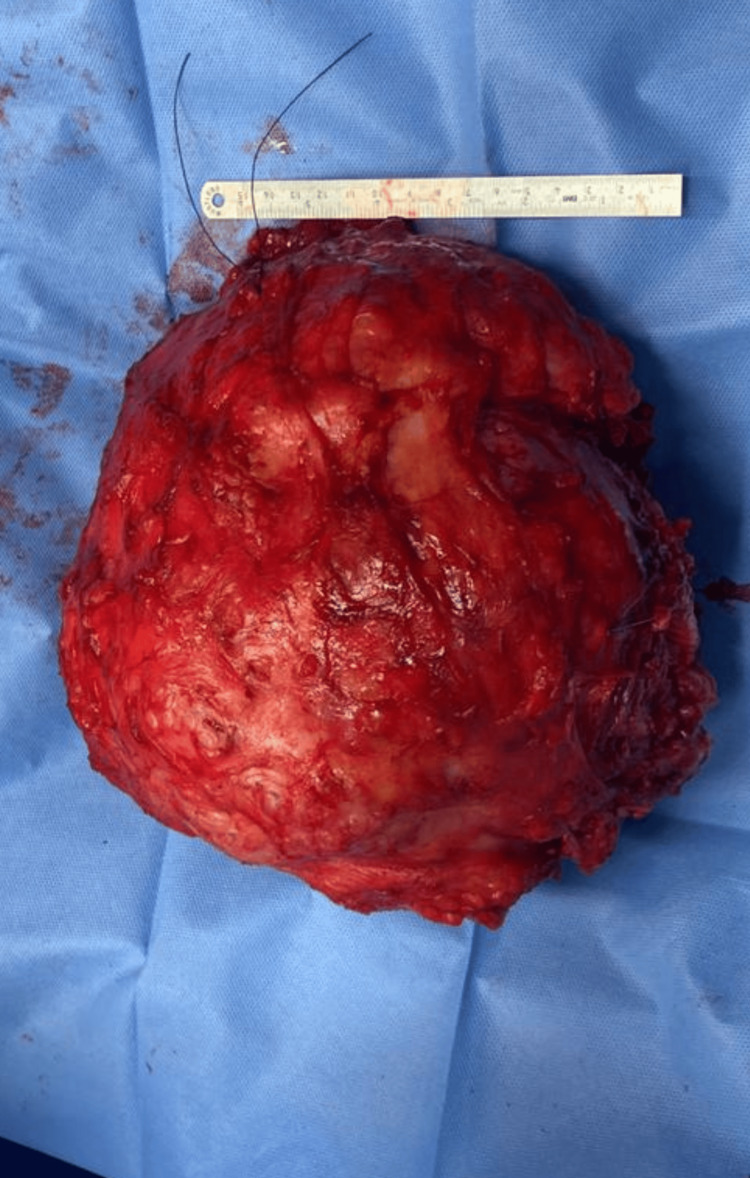
Intraoperative image of the tumor after the en bloc excision.

The excised scapular body was sent for intra-operative frozen section histopathology examination and was reported to have tumor-free margins. The tumor weighed approximately 4 kg and measured 26 × 24 × 17 cm. Macroscopically, the outer surface of the mass exhibited multiple gray nodular deposits on the superior, inferior, superficial, and deep surfaces (Figure 9).

**Figure 10:**
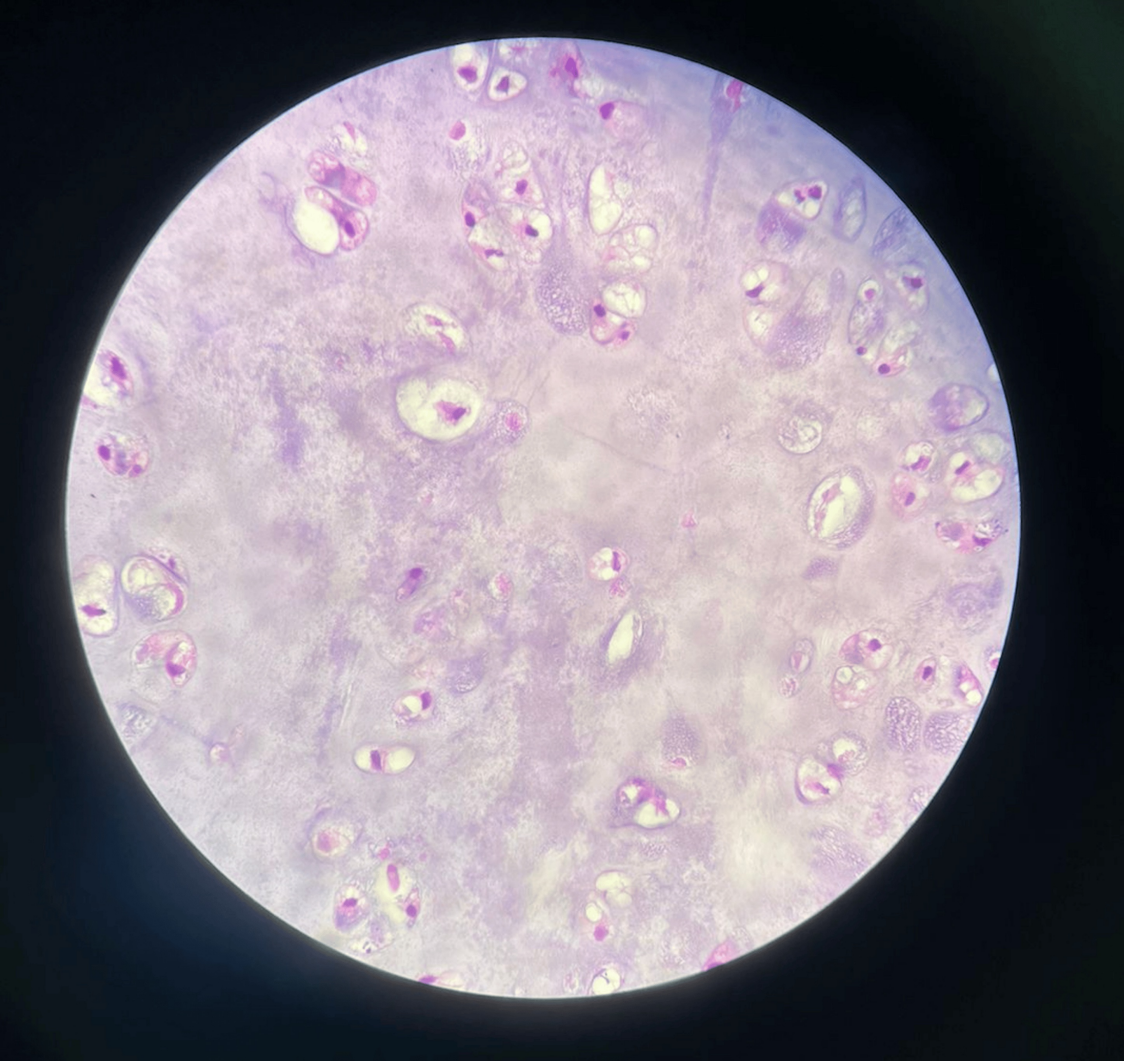
Postoperative histology slide of the tumor confirming the diagnosis of chondrosarcoma.

In order to preserve some function of the shoulder joint, the tendon of the latissimus dorsi muscle was sutured to the subscapularis tendon. The detached tendon of the inferior part of the trapezius was sutured to the tendons of the infraspinatus and teres minor muscles. The wound was then irrigated using copious amounts of sterile normal saline. Closure of the skin was then performed in layers after the placement of a drain. Postoperative tissue pathology confirmed the diagnosis of grade 2, low-grade chondrosarcoma, consistent with the radiological findings. The histological examination revealed multiple cores of white soft tissue, which are characteristic of this type of tumor (Figure 10).

However, despite follow-up visits at two, four, eight, and 10 weeks, the patient demonstrated poor adherence to the prescribed physical therapy regimen. This lack of compliance negatively affected his rehabilitation progress, as evidenced by his latest examination, which revealed limitations in the range of motion in all directions. The patient’s overall strength was assessed at three out of five (Table 1).

**Table 1:** Range of motion measurements of both shoulders. The first column shows the ROM in the operated shoulder. The second column shows the ROM in the contralateral normal shoulder. ABD: abduction; ER: external rotation; FF: forward flexion; IR: internal rotation; ROM: range of motion

	Active/passive ROM	Contralateral shoulder
FF	30/45	170
ABD	40/45	145
IR	Up to sacrum	L1
ER	45/45	80

**Figure 11:**
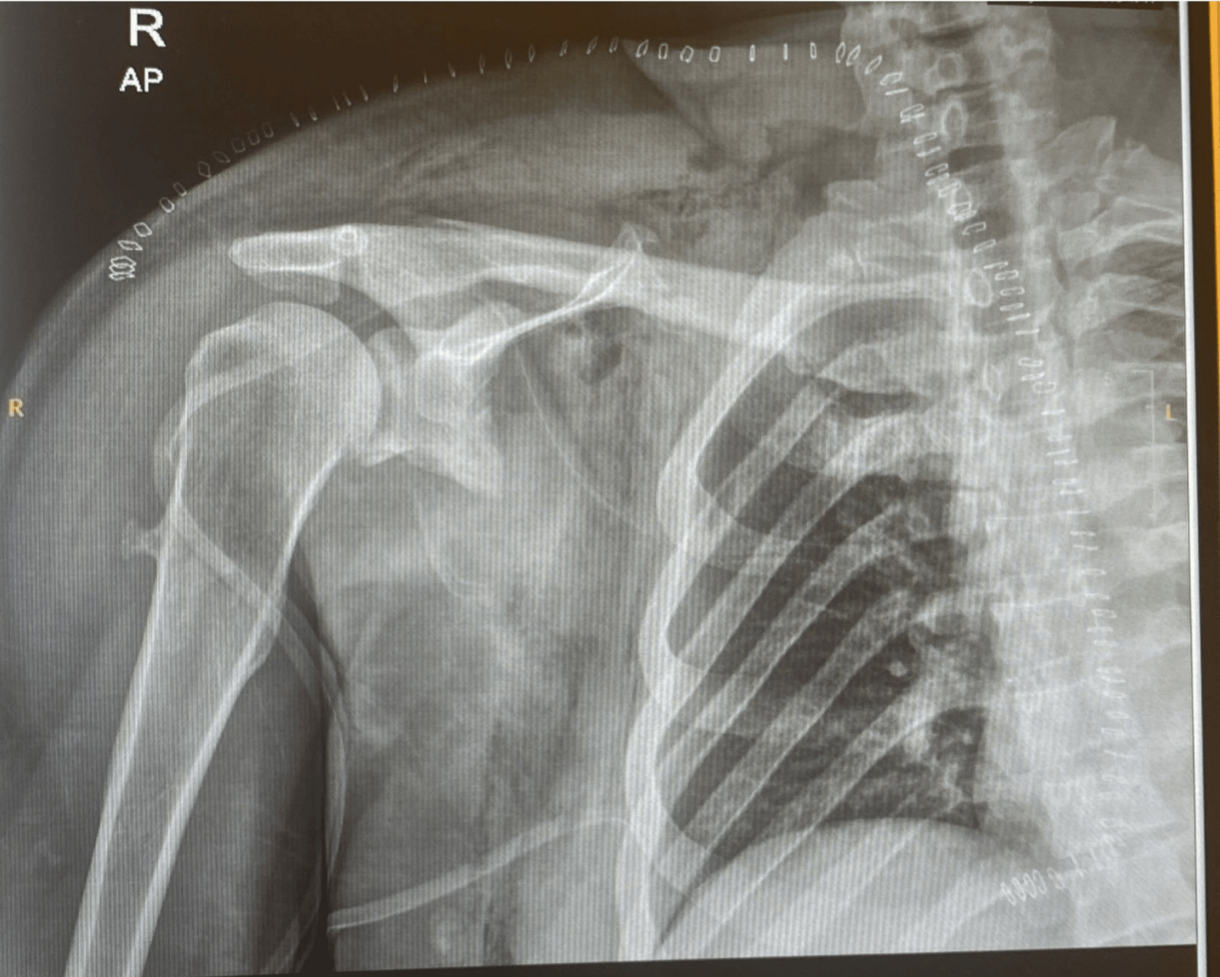
Postoperative X-ray of the right shoulder.

## Discussion

Chondrosarcoma is a malignant tumor characterized by cartilage production, accounting for 20-25% of all bone sarcomas [1]. It predominantly occurs in flat bones, with the scapula being a rare site of involvement, which is associated with a more favorable prognosis [2]. Typically, patients diagnosed with chondrosarcoma are middle-aged or elderly, exhibiting a slight male predominance [3]. The clinical presentation of this tumor is often nonspecific; patients may report a sudden increase in the size of a mass accompanied by pain.

The etiology of chondrosarcoma is primarily sporadic, although it can develop secondary to the malignant transformation of osteochondromas or enchondromas. Diagnosing chondrosarcoma relies on a comprehensive evaluation that includes clinical history, physical examination, and histological grading. Histologically, chondrosarcoma is classified into three grades (grade 1, 2, and 3) based on architectural features and cytological atypia [4,5]. The survival rates decrease with increasing tumor grade, with reported five-year survival rates of 90%, 81%, and 43% for grades 1, 2, and 3, respectively [5]. Conventional chondrosarcoma, primarily classified as grade 1, demonstrates survival rates of 80% to 90% and is less likely to metastasize due to its low malignancy potential. In contrast, approximately 70% of grade 3 chondrosarcomas exhibit hematogenous spread, typically to the lungs [5-7].

CT and MRI are the primary imaging modalities utilized in the diagnosis of chondrosarcoma. CT imaging typically reveals an expansive, osteolytic, destructive soft tissue mass with calcifications and irregular cystic low-density areas [8,9]. MRI, on the other hand, provides precise staging of medullary involvement and delineates the extent of the soft tissue mass, thereby guiding surgical resection. The typical imaging findings observed in our present case were consistent with these characteristics.

During surgical intervention, anatomopathological examination confirmed the presence of low-grade chondrosarcoma, characterized by low malignancy and a minimal risk of metastasis. Several noteworthy aspects of this case warrant attention: first, the asymptomatic nature of the patient’s presentation despite the presence of a large scapular mass, and second, the rarity of scapular chondrosarcoma, even in adult populations.

Historically, complete scapulectomy, along with complete upper limb amputation, was the primary treatment option for scapular chondrosarcoma. However, in recent years, advancements in sarcoma treatment have favored limb reconstruction surgery as the main modality, leading to improved survival rates. Effective surgical planning is critical, as it significantly influences the function of the affected limb. The successful reconstruction of bone and joint structures is heavily dependent on the restoration of surrounding soft tissues, regardless of the surgical method employed. Consequently, over 90% of malignant tumors can now be managed using limb salvage surgery [10].

In 1991, Malawer and colleagues developed a classification system for shoulder girdle resections based on anatomical location, extent of tumor involvement, and tumor grade [10]. For benign or low-grade malignant lesions, intra-articular resections (types I to III) are recommended. Conversely, high-grade sarcomas necessitate extra-articular resections (types IV to VI). The regional anatomy of scapular chondrosarcoma often allows for resections with negative margins, particularly when compared to tumors located in flat bone sites, such as the pelvis and chest wall, where achieving wide surgical margins can be challenging due to local invasion of adjacent structures [11,12].

In the presented case, the tumor was situated in the scapular region, allowing for marginal resection to preserve the function of the shoulder girdle and enhance the patient’s quality of life. Following the complete removal of the tumor, the patient regained 45º of flexion, 45º of abduction, and 45º of external rotation. He is currently undergoing a physiotherapy program, and no postoperative complications were reported.

However, it is essential to note that marginal surgical resection does not always guarantee negative margins. A study by Miura et al. demonstrated a high risk of local recurrence associated with inadequate surgical margins [13]. Similarly, research by Fiorenza et al. identified inadequate surgical margins and tumor size greater than 10 cm as independent risk factors for local recurrence [14].

Adjuvant radiation therapy can be utilized for patients with high-grade chondrosarcoma or those at increased risk of recurrence; however, it is generally not employed as an initial treatment modality. Chondrosarcoma typically exhibits a poor response to chemotherapy, but it may be effective as a neoadjuvant treatment, inhibiting tumor growth and progression [15]. Notably, chemotherapy has shown efficacy in mesenchymal chondrosarcoma, a rare and aggressive subtype characterized by small round blue cells. Delayed presentation of the tumor often results in unfavorable outcomes, as the tumor may be advanced at diagnosis and have metastasized to surrounding tissues.

## Conclusions

Chondrosarcoma of the scapula is considered a significantly rare type of bone tumor and is known for having a more favorable prognosis compared to chondrosarcomas located in the pelvis and chest wall. The prognosis for patients diagnosed with chondrosarcoma is significantly influenced by two main factors: the histological grade of the tumor and the extent of surgical resection. Specifically, low-grade chondrosarcomas are typically treated with surgical intervention, which aims to achieve wide margins and complete tumor removal. It is important to emphasize that the management of such tumors should be personalized after performing a thorough examination of the radiological images to determine the extent of the tumor, keeping in mind that free-margin excision of the whole tumor is more important than preserving the function of the shoulder joint. Moreover, early diagnosis and timely treatment of chondrosarcoma are vital, as they are correlated with improved prognoses and better survival rates. Healthcare professionals need to maintain a high index of suspicion for this rare tumor, especially in patients presenting with persistent pain or swelling in the scapular region. Overall, a multidisciplinary approach that includes orthopedic surgeons, oncologists, and radiologists is crucial for optimizing treatment strategies and improving patient outcomes in cases of chondrosarcoma.
